# A Novel Polyhalogenated Monoterpene Induces Cell Cycle Arrest and Apoptosis in Breast Cancer Cells

**DOI:** 10.3390/md17080437

**Published:** 2019-07-25

**Authors:** Menna El Gaafary, Susanne Hafner, Sophia J. Lang, Lu Jin, Omar M. Sabry, Carl V. Vogel, Christopher D. Vanderwal, Tatiana Syrovets, Thomas Simmet

**Affiliations:** 1 Institute of Pharmacology of Natural Products and Clinical Pharmacology, Ulm University, D-89081 Ulm, Germany; 2 Department of Pharmacognosy, College of Pharmacy, Cairo University, Cairo 11562, Egypt; 3 Department of Chemistry, 1102 Natural Sciences II, University of California, Irvine, CA 92697-2025, USA

**Keywords:** red algae, *Plocamium*, polyhalogenated monoterpenes, cell cycle, apoptosis, chick chorioallantoic membrane assay

## Abstract

Breast cancer is the most common cancer type and a primary cause of cancer mortality among females worldwide. Here, we analyzed the anticancer efficacy of a novel bromochlorinated monoterpene, PPM1, a synthetic analogue of polyhalogenated monoterpenes from *Plocamium* red algae and structurally similar non-brominated monoterpenes. PPM1, but not the non-brominated monoterpenes, decreased selectively the viability of several triple-negative as well as triple-positive breast cancer cells with different p53 status without significantly affecting normal breast epithelial cells. PPM1 induced accumulation of triple-negative MDA-MB-231 cells with 4N DNA content characterized by decreased histone H3-S10/T3 phosphorylation indicating cell cycle arrest in the G_2_ phase. Western immunoblot analysis revealed that PPM1 treatment triggered an initial rapid activation of Aurora kinases A/B/C and p21^Waf1/Cip1^ accumulation, which was followed by accumulation of polyploid >4N cells. Flow cytometric analysis showed mitochondrial potential disruption, caspase 3/7 activation, phosphatidylserine externalization, reduction of the amount polyploid cells, and DNA fragmentation consistent with induction of apoptosis. Cell viability was partially restored by the pan-caspase inhibitor Z-VAD-FMK indicating caspase contribution. In vivo, PPM1 inhibited growth, proliferation, and induced apoptosis in MDA-MB-231 xenografted onto the chick chorioallantoic membrane. Hence, *Plocamium* polyhalogenated monoterpenes and synthetic analogues deserve further exploration as promising anticancer lead compounds.

## 1. Introduction

Breast cancer is the most common cancer and the leading cause of cancer-related mortality among females world-wide accounting for 25% of all cancer cases and 15% of all cancer deaths in 2012 [1]. In the United States, breast cancer is considered to be the most frequently diagnosed cancer type and the second main cause of cancer death in women. By 2018, it is expected to account for 30% of all new cancer diagnoses and 14% of all estimated cancer deaths. Based on current incidence rates, 12.4% of US women will develop breast cancer during their lifetime [2].

Triple-negative breast cancer (TNBC) is characterized by the lack of expression of estrogen and progesterone receptors as well as the absence of overexpression of human EGF receptor 2 [3,4]. TNBC more frequently affects younger premenopausal women, follows an aggressive clinical course, and metastasizes to critical visceral organs, such as lung, liver, and brain. TNBC is associated with a higher risk of recurrence in the first years after completion of the therapy and shorter median survival time of patients [3,4,5]. In recent years, the development of highly selective, molecularly tailored agents has shown considerable success in the treatment of endocrine or HER2-driven breast cancer. Unfortunately, such advancements have proven elusive for TNBC due to the lack of an established receptor target. 

Many species of red algae (*Rodophyta*) synthesize a wealth of halogenated natural products, many of them have been reported to possess promising biological activity, including unique cytotoxic profiles [6,7]. These halogenated monoterpenes bear mostly chiral vicinal dihalide motifs, with the majority being bromochlorinated terpenes [8,9]. They could be either cyclic or acyclic and have been confined to the red algae genera *Plocamium* and *Chondrococcus* [6,7,10]. 

The class of polyhalogenated monoterpenes has sparked great interest due to the promising profile of selective cytotoxic activity of halomon against cell lines derived from highly chemoresistant solid human tumors and to the unique yet-unknown mechanism of its cytotoxic action [7,11,12]. The pharmacokinetics of halomon in mice revealed its marked and persistent accumulation in fat tissues as a result of its lipophilic nature and multiple halogens in its structure [11]. For that reasons, such molecules might hold promise for the treatment of malignancies that grow in adipocyte-rich environment, such as breast cancer. However, the clinical application for this category of compounds as anticancer agents is hampered due to paucity of mechanistic information [13,14] and a complex purification procedure from reliable natural source or selective stereo-controlled synthesis [8,9,12,14].

Total synthesis of marine-derived analogues may ensure a sustainable and reliable supply of the active polyhalogenated monoterpenes, and might also establish the basis for generating a wide range of analogues to expedite the appraisal of structure activity relationship of this novel class of cytotoxic agents for their further development as oncological drugs. Over the past 25 years, many attempts were made to synthesize halomon and its analogues [8,15,16,17] with the recent work by Burns and co-workers standing out for its high efficiency and stereoselectivity [8]. However, despite the reported cytotoxic and antimalarial activities of the acyclic *Plocamium* polyhalogenated monoterpenes [18,19,20,21,22,23], there were no reports of their synthesis until we disclosed a short and scalable strategy for the enantioselective and divergent synthesis of many of these highly inter-halogenated monoterpenes [9]. In our previous study [9], four different naturally occurring *Plocamium* polyhalogenated monoterpenes (PPM), one geometrical isomer of one of these natural products (called PPM1 in this study), and two enantiomeric analogues (PPM2 and PPM3) were synthesized. PPM1 is the *E*-isomer (at the chlorinated alkene) of a known natural product, and, many closely related compounds with the same alkene configuration have been isolated from natural sources. Therefore, it is highly likely that PPM1 is also synthesized in nature. So far, the cytotoxic potential of only 3 natural compounds were analyzed using different solid cancer cell lines, and PPM1-3 were not among them. All the compounds analyzed showed selectivity for solid cancer cell lines with some difference in the selectivity profile. Among the tested natural polyhalogenated monoterpenes, the one with the highest potency against the HCT-116 colon carcinoma cell line had an IC_50_ value of 4.2 µM [9]. The cytotoxic potential of PPM1—a compound presumed to be naturally occurring, but not reported—or the synthetic marine-derived analogues PPM2 and PPM3 have not been investigated yet. These latter compounds have the *syn*-dichloride arrangement and are thus diastereomeric with respect to PPM1 (*anti*-dichloride).

Considering the urgent need for effective and safe chemotherapeutic strategies for aggressive triple-negative breast cancer, we focused on polyhalogenated monoterpenes as potential oncological lead compounds. A preliminary screen was performed to evaluate the cytotoxic potential of the unanalyzed monoterpenes and the underlying cytotoxic mechanism by using the highly aggressive triple-negative MDA-MB-231 breast cancer cell line.

## 2. Results

### 2.1. PPM1 Is Cytotoxic for Human Breast Cancer Cells with Little Effect on Normal Human Mammary Epithelial Cells

**Figure 1:**
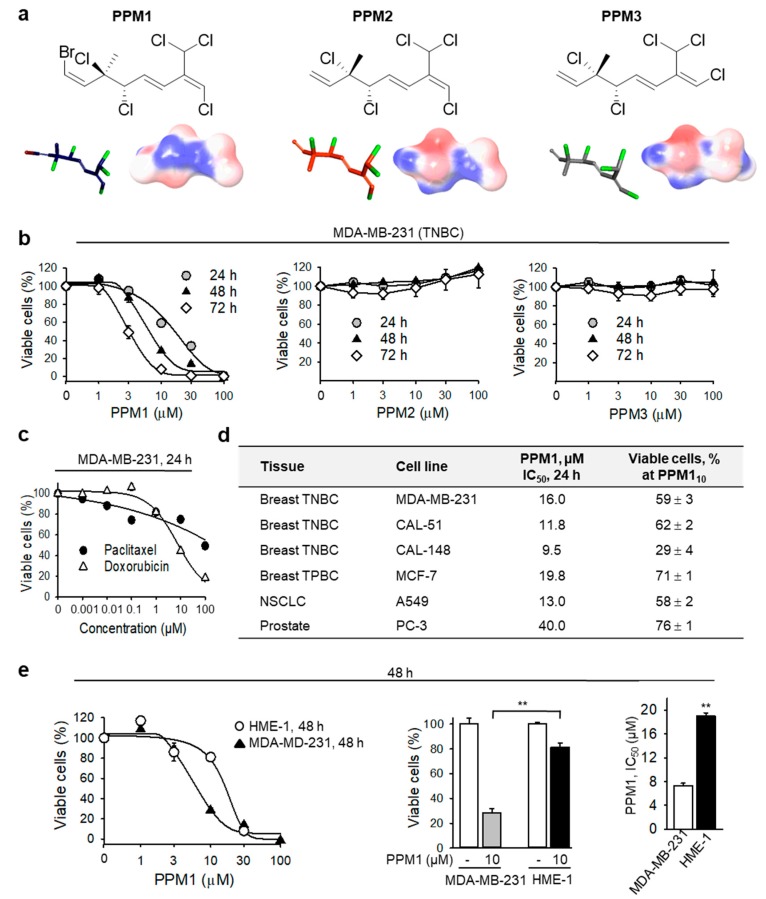
PPM1, but not PPM2 or PPM3, is selectively cytotoxic to breast cancer cells. (**a**) Structures and three-dimensional electrostatic potential models of compounds PPM1, PPM2, and PPM3. The negatively charged regions are red and the positively charged regions are blue. PPM1 (violet carbon sticks), PPM2 (orange carbon sticks), and PPM3 (grey carbon sticks) (bromine, red; chlorine, light green; hydrogen atoms omitted for clarity). (**b**) MDA-MB-231 cells were treated with the marine-derived synthetic polyhalogenated monoterpene analogues for the indicated times. Cell viability was analyzed by a XTT cell viability assay. (**c**) MDA-MB-231 cells were treated with different concentrations of doxorubicin or paclitaxel for 24 h and analyzed as in (**b**). (**d**) Different cancer cell lines were treated with PPM1 for 24 h and analyzed as in (**b**). NSCLC, non-small cell lung cancer; PPM1_10_ shows number of viable cells after treatment with 10 µM PPM1 for 24 h. (**e**) Normal human primary mammary epithelial HME-1 cells are relatively resistant to PPM1. Cells were treated for 48 h and analyzed as in (**b**). Statistical analysis was performed by using the Newman-Keuls test. Data are mean ± SEM, *n* = 3, ** *p* < 0.01. All data are mean ± SEM, *n* = 3.

Among three compounds investigated, only the brominated monoterpene PPM1, but not PPM2 or PPM3 (Figure 1a,b) decreased the viability of MDA-MB-231 TNBC cells in a concentration- and time-dependent manner with IC_50_ values of 16 ± 2.2 µM, 7.3 ± 0.4 µM, and 3.3 ± 0.5 µM after 24 h, 48 h, and 72 h, respectively. MDA-MB-231 are highly aggressive triple-negative breast cancer cells. The first line treatment for patients with triple-negative breast cancer are the non-halogenated anthracyclines and taxane-based adjuvant regimens [24]. The anthracycline doxorubicin (Adriamycin^TM^) inhibited the cell viability with an IC_50_ of 7.8 ± 0.7 µM after 24 h. By contrast, after the same treatment time, paclitaxel (Taxol^TM^) at 100 nM decreased the percentage of viable MDA-MB-231 cells by only 20%, without any further decline in cell viability by paclitaxel concentrations up to 100 µM (Figure 1c). Furthermore, PPM1 exerted toxicity on the additional TNBC cell lines CAL-51 and CAL-148, and the triple-positive breast cancer (TPBC) cell line MCF-7, as well as on non-small cell lung cancer (NSCLC) and prostate cancer cells (Figure 1d). CAL-51 and MCF-7 express wild type p53, whereas MDA-MB-231 and CAL-148 are p53 mutants [25] indicating that PPM1 exhibits toxicity to breast cancer cell lines with different p53 status. Notably, PPM1 appeared to have a small effect (~20% inhibition) on the viability of normal human mammary epithelial HME-1 cells (Figure 1e). Analysis of PPM1 toxicity by using normalized growth inhibition (GR) values, which consider also the drug impact on cell proliferation [26], revealed GR_50_ values of PPM1 for MDA-MB-231 and MCF-7 of 5.0 and 7.1 µM, respectively. Hence, MDA-MB-231 is slightly more sensitive to PPM1 compared to MCF-7, which correlates with the respective IC_50_ values after 24 h (Figure 1d). The PPM1 GR_50_ for HME-1 cells with 13.1 µM is higher than those for the breast cancer cells pointing to a relative selectivity of the drug for cancer cells. GR_max_ for all cells were negative indicating that PPM1 exerts cytotoxic rather than cytostatic effect.

### 2.2. PPM1 Induces Cell Cycle Arrest in Triple-Negative MDA-MB-231 Breast Cancer Cells

**Figure 2:**
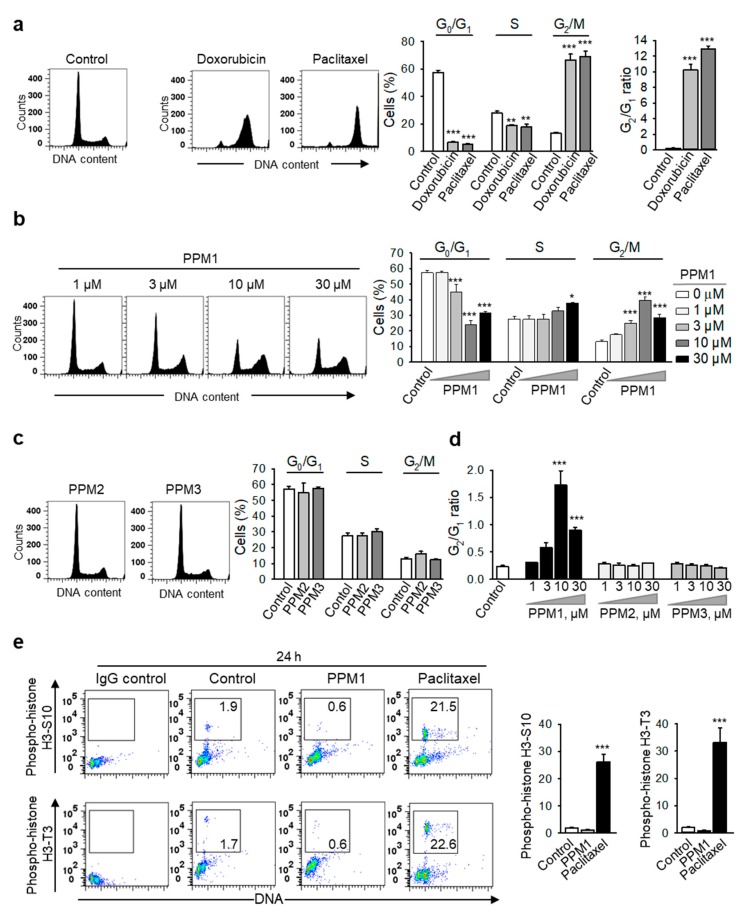
PPM1, but not PPM2 or PPM3, induces a concentration-dependent accumulation of breast cancer cells in the G_2_ phase. MDA-MB-231 cells were treated with doxorubicin (100 nM) or paclitaxel (100 nM) (**a**), different concentrations of PPM1 (**b**), PPM2 or PPM3 (each 30 µM) (**c**) for 24 h. DNA was stained with propidium iodide and cells were analyzed by using flow cytometry. Representative histograms are shown on the left. Bar graphs on the right show the percentages of MDA-MB-231 cells in each phase of the cell cycle. (**d**) Graph shows the mean G_2_/G_1_ ratio of cells treated as in (**b**) and (**c**). (**e**) Different to paclitaxel, PPM1 did not increase the mitotic p-H3 cell population. MDA-MB-231 cells were treated with 10 µM PPM1 or 100 nM paclitaxel for 24 h, fixed, permeabilized with methanol, stained with antibodies against phosphorylated histone H3-S10 and H3-T3 and propidium iodide, and analyzed by flow cytometry. Representative dot blots are shown. Graphs show percentage of mitotic p-H3-T3^+^ cells. Statistical analysis was performed by using the Newman-Keuls test. Data are mean ± SEM, *n* = 3, * *p* < 0.05, ** *p* < 0.01, *** *p* < 0.001.

Based on the differential cytotoxic potential of PPM1 towards the malignant MDA-MB-231 cells compared to normal human mammary epithelial cells, we next addressed the molecular mechanism of PPM1 cytotoxicity in comparison to standard chemotherapeutic agents. Doxorubicin- or paclitaxel-treated MDA-MB-231 cells exhibited a pronounced accumulation of cells in the G_2_/M phase (4N) with a significant reduction of cells in the G_0_/G_1_ (2N) and S phases (Figure 2a). Similarly, cell-cycle analysis of cells treated with PPM1 for 24 h revealed a significant concentration-dependent accumulation of the malignant cells in the G_2_/M phase (25 and 40% for 3 and 10 µM PPM1, respectively) as compared to control (13%). Concomitantly, PPM1 induced a significant reduction of cells in the G_0_/G_1_ phase of the cell cycle, which accounted for 45% (3 µM PPM1) and 24% (10 µM PPM1) relative to controls (57.2%) (Figure 2b). As expected and at variance to PPM1, compounds PPM2 and PPM3 showed basically no effect on the cell-cycle progression of MDA-MB-231 cells (Figure 2c,d).

Detailed analysis of the cell cycle distribution of MDA-MB-231 cells revealed that within 24 h, PPM1 treatment triggered no increase, but even a decrease in the proportion of cells positive for phosphorylated histone H3-S10 and H3-T3 (Figure 2e), mitotic markers. H3 phosphorylation is initiated during chromosome condensation in the prophase of mitosis with peak levels in the metaphase [27]. However paclitaxel, which is known to induce mitotic arrest [28], significantly increased the amounts of p-histone H3-S10^+^ and H3-T3^+^ cells after 24 h (Figure 2e). Hence, PPM1 induced accumulation of cancer cells in the G_2_ phase of the cell cycle.

### 2.3. PPM1-Treated MDA-MB-231 Cells Exhibit Early Transient Activation of Aurora Kinases A/B/C in Triple-Negative MDA-MB-231 Breast Cancer Cells

**Figure 3:**
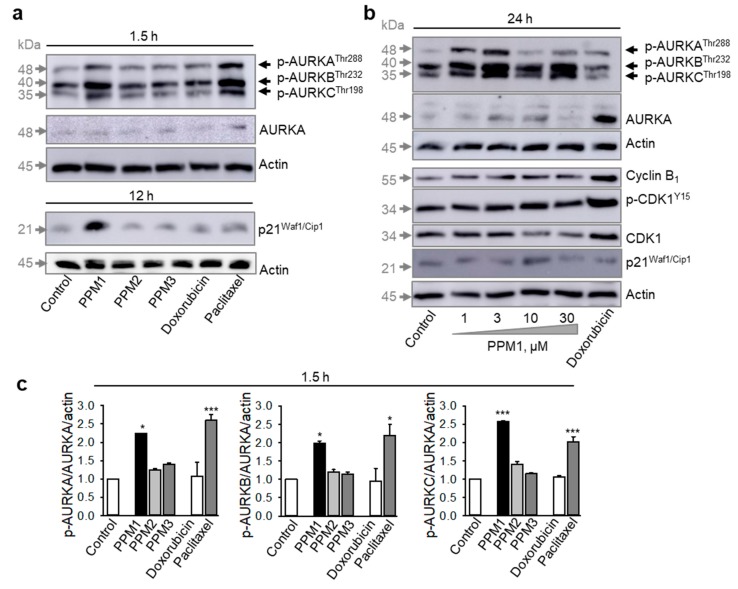
PPM1-treated cells exhibit activation of Aurora kinases and expression of p21^Waf1/Cip1^. (**a**) MDA-MB-231 cells were treated with the indicated polyhalogenated monoterpenes (10 µM each), doxorubicin (100 nM), or paclitaxel (100 nM) for 1.5 or 12 h and were analyzed by Western immunoblotting. For protein loading control, membranes were reprobed with anti-actin antibody. (**b**) MDA-MB-231 cells were treated with different concentrations of PPM1 or doxorubicin (100 nM) for 24 h and analyzed by western immunoblotting. Representative membranes are shown. (**c**) Quantification of the expression of phospho-Aurora kinases A, B, C in cells treated as in (**a**). Statistical analysis was performed by using the Newman-Keuls test, * *p* < 0.05, *** *p* < 0.001 vs. control.

Already within 90 min of treatment, PPM1 and the mitotic poison paclitaxel, but neither doxorubicin, nor PPM2 or PPM3, induced a dramatic increase in the phospho-Aurora A^T288^/Aurora B^T232^/Aurora C^T198^ levels (Figure 3a). At a later point of time, PPM1 still induced an increase of the activity of Aurora kinases A/B/C with a maximum response after treatment with the subcytotoxic PPM1 concentrations, 1 and 3 µM, and a decline in the phosphorylation levels of Aurora kinases could be detected at cytotoxic PPM1 concentrations (Figure 3b). Doxorubicin had a little effect on the activity of Aurora kinases A/B/C, but treatment with doxorubicin profoundly increased the total level of Aurora kinase A (Figure 3b) apparently due to enhanced accumulation of cells in the S/G_2_ phase, which is characterized by high expression of Aurora kinase proteins [29]. Different to doxorubicin, treatment of MDA-MB-231 cells with PPM1 for 24 h showed no significant changes in the expression of cyclin B_1_ and CDK1, and relatively low CDK1 activation as evident from an inhibitory phosphorylation on Y15 (Figure 3b). In addition, the activation of Aurora kinases observed with a cytotoxic PPM1 concentration of 10 µM after 90 min treatment was normalized to about control levels 24 h later (Figure 3a,b). Next, we analyzed the effect of PPM1 on the expression of the cyclin-dependent kinase inhibitor 1, p21^Waf1/Cip1^. 

Treatment of MDA-MB-231 cells with different polyhalogenated monoterpenes, doxorubicin, or paclitaxel for 12 h revealed that PPM1 was the only compound to induce a robust though transient increase in p21^Waf1/Cip1^ expression (Figure 3a,b) at the cytotoxic concentration of 10 µM. The induction of p21^Waf1/Cip1^ is a form of cellular response to stress, which might play a role in PPM1 toxicity. 

### 2.4. PPM1 Induces Formation of Polyploid Cells in Triple-Negative MDA-MB-231 Breast Cancer Cells

**Figure 4:**
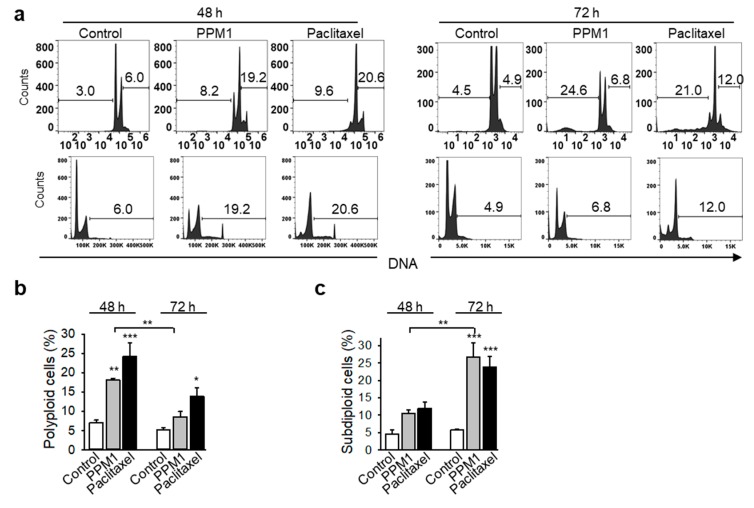
PPM1 induces formation of polyploid breast cancer cells. (**a**) MDA-MB-231 cells were treated with PPM1 (10 µM) or paclitaxel (100 nM) for 48 or 72 h, and DNA was stained with propidium iodide. Polyploid and subdiploid cells were analyzed by using flow cytometry. Representative histograms in logarithmic and linear scales are shown. (**b**) and (**c**) graphs show the percentages of polyploid and apoptotic subdiploid cells, respectively. All data are mean ± SEM, *n* = 3. Statistical analysis was performed by using the Newman-Keuls test, * *p* < 0.05, ** *p* < 0.01, *** *p* < 0.001.

PPM1 induced formation of a polyploid cell population after 48 h of treatment (Figure 4a,b). The polyploid cell population, which was formed in PPM1-treated cells after 48 h, was significantly reduced in PPM1-treated cells after 72 h, whereas the apoptotic subG_1_ population increased (Figure 4a–c).

### 2.5. PPM1 Induces Mitochondrial Membrane Dissipation in Triple-Negative MDA-MB-231 Breast Cancer Cells

**Figure 5:**
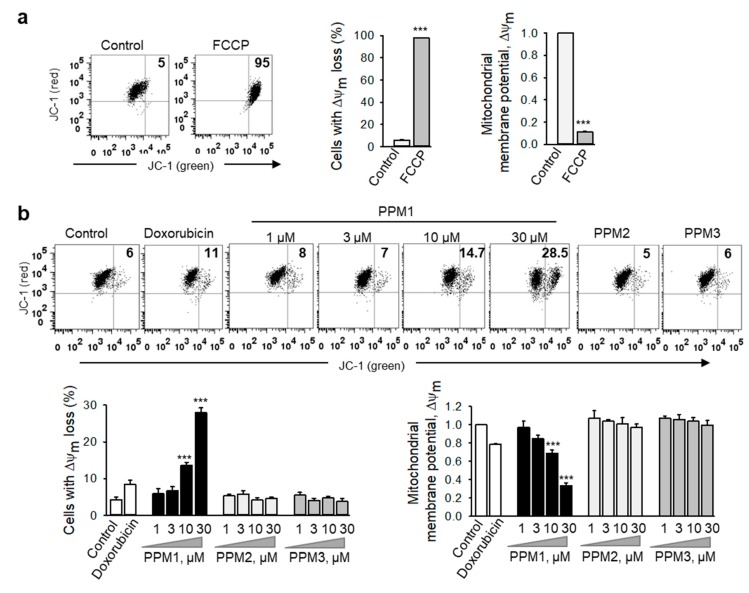
PPM1 affects the mitochondrial integrity in breast cancer cells. (**a**) MDA-MB-231 cells treated with the respiratory uncoupler, FCCP (50 µM) for 6 h were used as positive control. (**b**) MDA-MB-231 cells were treated with doxorubicin (100 nM), different concentrations of PPM1, PPM2 or PPM3 (each at 30 µM) for 24 h. The mitochondrial membrane potential was analyzed flow cytometrically by using JC-1 dye. Representative dot plots are shown. Figures (upper right square) show number of cells with the loss of mitochondrial membrane potential (ΔΨm). ΔΨm was measured as red/green JC-1 fluorescence intensity ratio. Graphs demonstrate the percentages of MDA-MB-231 cells with depolarized mitochondria and the loss of ΔΨm in treated cells. Statistical analysis was performed by using the Newman-Keuls test. All data are mean ± SEM, *n* = 3, *** *p* < 0.001 vs. control.

Blockage of cell division prevents cell proliferation but does not necessarily kill cells. Since disruption of mitochondrial function is a critical early event in cell commitment to apoptosis [30], we have analyzed the mitochondrial membrane integrity by using the lipophilic cationic JC-1 dye (Figure 5). The dye specifically accumulates inside the negatively-charged energized mitochondria in a potential-dependent manner to form red fluorescence aggregates, whereas in damaged mitochondria, it becomes dispersed in the cytoplasm leading to a fluorescence emission shift from red to green. Among the analyzed polyhalogenated compounds, only PPM1 induced a significant concentration-dependent loss in the mitochondrial membrane potential ΔΨm in MDA-MB-231 cells characterized by a shift of red-to-green fluorescence intensity and an increase in the percentages of cells with deenergized mitochondria similar to the effects seen with the potent mitochondrial oxidative phosphorylation uncoupler, FCCP (Figure 5a–c).

### 2.6. PPM1 Induces Apoptosis in Triple-Negative MDA-MB-231 Breast Cancer Cells

**Figure 6:**
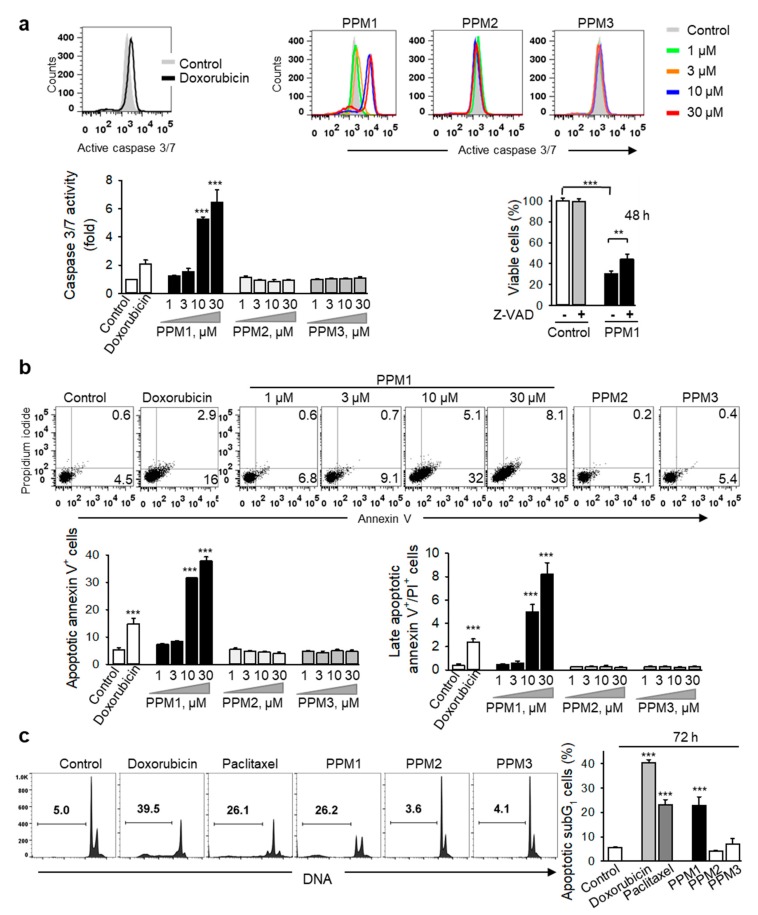
PPM1 induces apoptosis in breast cancer cells. (**a**) PPM1 induces activation of caspase 3/7 in breast cancer cells, which is partly responsible for its cytotoxicity. MDA-MB-231 cells were treated with different concentrations of the polyhalogenated monoterpenes or doxorubicin (100 nM) for 24 h, incubated with the caspase 3 substrate Z-DEVD-R110, and the fluorescent cleavage product was analyzed by using flow cytometry. Representative histograms are shown. Left hand graph shows the caspase 3/7 activity expressed as fold increase compared to the control. Right hand graph shows viability of MDA-MB-231 cells pretreated with the pancaspase inhibitor Z-VAD-fmk (50 µM) for 2 h, then treated with PPM1 (10 µM) for further 48 h, and analyzed using XTT assay. (**b**) MDA-MB-231 cells were treated for 48 h as described in (**a**), stained with FITC-annexin V/propidium iodide and analyzed by using flow cytometry. Representative dot plots are shown; PPM2 or PPM3 (each at 30 µM). Graphs represent quantitative data of the distribution of apoptotic cells based on annexin V/PI co-staining. (**c**) PPM1 induces DNA fragmentation in breast cancer cells. MDA-MB-231 cells were treated for 72 h with either PPM1, PPM2, PPM3 (10 µM, each), 100 nM doxorubicin, or 100 nM paclitaxel. DNA of the treated cells was stained with propidium iodide and analyzed by flow cytometry. Representative histograms are shown. Numbers indicate percentage of subdiploid apoptotic cells (subG_1_). Graph shows the percentages of subG_1_ cells. All data are mean ± SEM, *n* = 3, ** *p* < 0.01, *** *p* < 0.001 vs. control.

Mitochondria play an integral role in the activation of the caspase cascade in the intrinsic apoptotic signaling pathway [30]. Caspase 3 and 7 are the best characterized executioner caspases that when activated, cleave a restricted set of target proteins to produce the morphological and biochemical features associated with canonical apoptosis [31]. Treatment of MDA-MB-231 cells with PPM1 for 24 h induced a concentration-dependent activation of caspase 3/7 (Figure 6a). To confirm the contribution of caspases in PPM1-induced cell death, we conducted an inhibitory assay with the pan-caspase inhibitor Z-VAD-FMK. Indeed, Z-VAD-FMK partly restored the cell viability suggesting that caspase-dependent-apoptosis plays a role in the cytotoxic effect of PPM1 (Figure 6a).

Loss of phospholipid asymmetry resulting in exposure of phosphatidylserine residues at the cell surface is a caspase-dependent process [31]. Analysis of the cell membrane asymmetry and integrity using annexin V/propidium iodide co-staining used to sort early and late apoptotic cells by flow cytometry revealed that treatment of MDA-MB-231 cells with PPM1 for 48 h resulted in a significant concentration-dependent increase in the percentage of annexin V-positive cells (Figure 6b).

In the apoptotic signaling cascade, DNA fragmentation is a late caspase-dependent event and a universal biochemical hallmark of apoptosis [31]. Not surprisingly, treatment of MDA-MB-231 cells with PPM1 for 72 h increased the percentage of cells with subG_1_ DNA contents, similar to that of doxorubicin and paclitaxel, confirming induction of apoptotic cell death by PPM1 in the highly aggressive MDA-MB-231 breast cancer cells (Figure 6c). By contrast, neither PPM2- nor PPM3-treated cells exhibited any of above mentioned signs of apoptosis (Figure 6a–c). 

### 2.7. PPM1 Inhibits Proliferation and Induces Apoptosis in Triple-Negative MDA-MB-231 Xenografts

**Figure 7:**
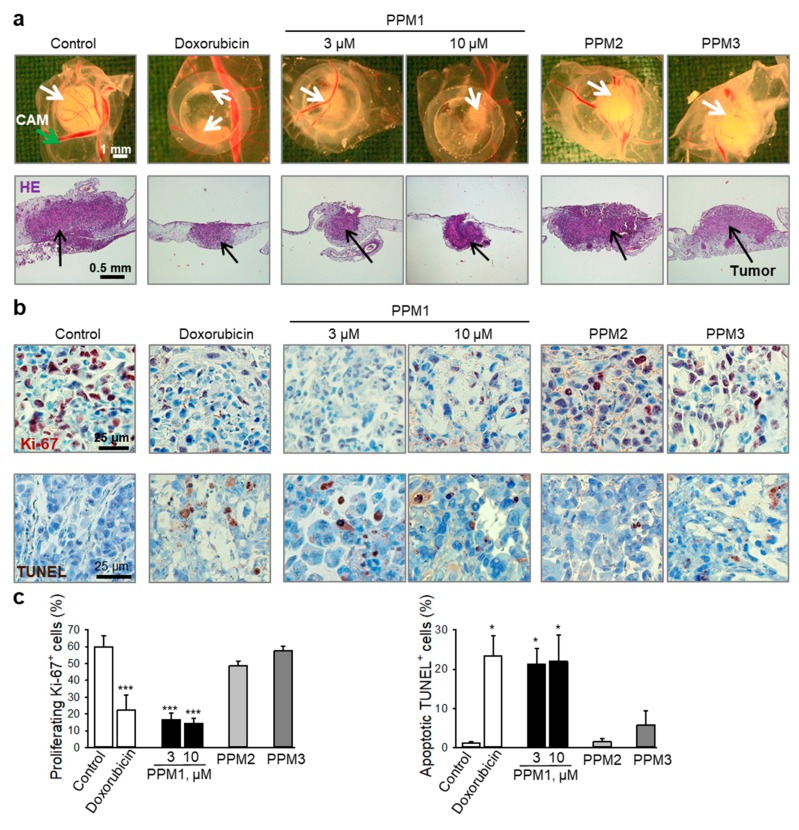
PPM1 induces apoptosis and inhibits growth of breast cancer xenografts in vivo. MDA-MB-231 cells were grafted onto the chorioallantoic membrane (CAM) of fertilized chick eggs. Next day, the tumors were topically treated with PPM1 (3 or 10 µM), PPM2 (10 µM), PPM3 (10 µM), or doxorubicin (10 µM) for 3 consecutive days. (**a**) Representative pictures from 4 biological replicates are shown. Upper panel: Pictures of tumor xenografts taken immediately after extraction. White arrows—tumors. Lower panel: hematoxylin and eosin staining (HE) after histological preparation, original magnification 50×. Black arrows—tumors. (**b**) Upper panels: Immunohistochemical analysis of tumor cell proliferation using nuclear Ki-67 antigen (proliferation marker, dark violet nuclear stain). Nuclei were counterstained with hematoxylin (blue). Original magnification 200X. Lower panels: TUNEL staining (TdT, dark brown) for detection of cells with fragmented DNA as apoptosis marker. Original magnification 200X. (**c**) Graphs show quantification of tumor cell proliferation and apoptosis. Statistical analysis was performed by using the Newman-Keuls test, *n* = 3–4, * *p* < 0.05, *** *p* < 0.001 vs. control.

The in vitro cytotoxic and apoptotic properties of PPM1 were further assessed in an in vivo model using MDA-MB-231 xenografts on the chorioallantoic membrane of fertilized chick eggs (CAM). Immunohistochemical analysis revealed that PPM1 inhibited the expression of the nuclear antigen Ki-67, used as a marker for growth and proliferation of the tumor xenografts, and induced DNA strand breaks, a sign of induction of apoptosis, in the highly aggressive MDA-MB-231 breast cancer xenografts (Figure 7a,b). Upon examination of the embryos, neither embryo death nor any signs of overt systemic toxicity associated with PPM1 treatment could be detected.

## 3. Discussion 

Although cytotoxic chemotherapy including paclitaxel and doxorubicin are widely used for the treatment of advanced human cancer, drug resistance and adverse effects are two major clinical hurdles limiting sustainable therapeutic efficacy. 

The marine environment has proven to be a prolific source of compounds with interesting biological activities [6]. Among marine natural products, terpenoids have provided and still represent an interesting pool of therapeutic bioactive molecular architectures that have demonstrated effectiveness against several human cancer cells [6,7,11,12,13]. By the development of a unique strategy, different derivatives of *Plocamium* monoterpenes were synthesized including bromochlorinated PPM1 and chlorinated PPM2 and PPM3 [9]. The cytotoxic potential of these polyhalogenated monoterpene analogues has not yet been reported. PPM1 is the only brominated compound tested in the current study, and its three-dimensional electrostatic potential and electron charge density distribution is noticeably different from that of the non-brominated diastereomers PPM2 and PPM3. These discrepancies in molecular structure, stereochemistry, and charge distribution correlate to a difference in biological outcomes and in distinct mechanistic effects. Thus, only PPM1 exhibited toxicity against MCF-7 TPBC cells and the highly aggressive TNBC cells. Notably, PPM1 exhibited higher IC_50_ and GR_50_ values and, thus, less toxicity to normal human mammary epithelial cells besides low systemic toxicity in vivo. This points to a selectivity of PPM1 against cancer cells favoring its potential use as anticancer therapeutic regimen with fewer adverse drug reactions than conventional cytotoxic chemotherapy. 

A fundamental feature of cancer is the cell-cycle perturbation associated with uncontrolled proliferation and genomic and chromosomal instabilities. Therefore, targeting the cell cycle in cancer cells could hold benefit in the management of malignant diseases [32]. In this regard, we show that PPM1, similar to the cyclic polyhalogenated monoterpene, mertensene [13], caused accumulation of a substantial cell fraction in the G_2_/M phase already by 24 h post treatment. We show that at cytotoxic concentrations, PPM1-treated cells exhibited a rapid transient activation of Aurora kinases after 1.5 h, a surrogate marker for G_2_/M arrest, the level of which was reduced to that of the control after 24 h. Importantly, PPM1 decreased the proportion of MDA-MB-231 cells positive for phospho-histone H3-T3, indicating G_2_ arrest_._ Similar to the mitotic inhibitor paclitaxel, PPM1 treatment increased the percentage of polyploid cells (48 h post-treament), which might be due to the endoreplication cycle between S and a gap phase [33]. Such cells, similar to PPM1-treated cells, display no signs of mitosis [33]. At a later point of time (72 h), the polyploid cell population in PPM1-treated cells was significantly reduced, whereas the apoptotic subG_1_ population increased, indicating cell death. 

Interestingly, the induction of p21^Waf1/Cip1^ in MDA-MB-231 cells occurred at a PPM1 concentration inducing repression of Aurora kinase A/B/C activity, cell cycle arrest, and cell death indicating that in PPM1-treated cells, p21^Waf1/Cip1^ might have a role beyond its function as cell cycle inhibitor. Indeed, under certain cellular stresses, p21^Waf1/Cip1^ might also promote apoptosis through both p53-dependent and p53-independent mechanisms [34]. Particularly, mitochondrial membrane depolarization was shown to be essential for the p21^Waf1/Cip1^-mediated apoptosis [35]. Consistent with these previous reports, PPM1 induced mitochondrial depolarization and apoptosis in MDA-MB-231 cells as demonstrated by several biochemical markers of apoptosis, such as caspase 3 activation, loss of phospholipid asymmetry of the cell membrane, and late DNA fragmentation. Our in vitro study was further supported by in vivo analysis, where PPM1 exerted antitumor activity as efficient as doxorubicin against MDA-MB-231 xenografts through inhibition of proliferation and the induction of apoptosis.

In summary, our study provides novel mechanistic information on the polyhalogenated monoterpene analogues, PPM1, PPM2, and PPM3. Only PPM1 exhibited cytotoxic activity against breast cancer cells with different p53 status. The underlined mechanistic studies in MDA-MB-231 cells point to G_2_ cell cycle arrest, which involves activation of Aurora kinase A/B/C and a decrease in the proportion of cells positive for phosphorylated histone H3-T3. Subsequently, the cell cycle arrest was followed by induction of apoptosis. The inception of apoptosis might occur due to induction of p21^Waf1/Cip1^ and dissipation of the mitochondrial membrane potential in cancer cells that specifically drives malignant cells to apoptotic cell death. Thus, PPM1 is an interesting synthetic analogue of bromochlorinated *Plocamium* monoterpenes that merits further studies for potential clinical applications in oncology. 

## 4. Materials and Methods

### 4.1. Reagents

Three analogues of *Plocamium* polyhalogenated monoterpenes: (1*E*,3*E*,5*S*,6*R*,7*Z*)-8-bromo-1,5,6-trichloro-2-(dichloromethyl)-6-methylocta-1,3,7 triene (PPM1), (1*E*,3*E*,5*S*,6*S*)-1,5,6-trichloro-2-(dichloromethyl)-6-methylocta-1,3,7-triene (PPM2) and (1*Z*,3*E*,5*S*,6*S*)-1,5,6-trichloro-2-(dichloro- methyl)-6-methylocta-1,3,7-triene (PPM3), were synthesized using glyceraldehyde acetonide as a chiral pool precursor to develop an enantioselective and divergent synthesis strategy [9]. The synthesized compounds were purified to chemical homogeneity and their structure and conformation were investigated by various physicochemical approaches, such as optical rotation, retention factor, IR, ^1^H- and ^13^C-NMR spectroscopy, and mass spectrometry [9]. Stock solutions of the studied monoterpenes were prepared at concentrations of 20 mM in dimethyl sulfoxide and stored at -80 °C to maintain their chemical stability. Just before the biological experiments, serial dilutions from the stock solutions were prepared and further diluted with medium (DMEM, Life Technologies, Carlsbad, CA, USA) supplemented with 1% heat-inactivated fetal calf serum. Three-dimensional electrostatic potential charge distribution for the polyhalogenated monoterpenes was analyzed in the Jaguar module of Schrödinger Suite (New York, NY, USA) and 3D structures were created by Discovery Studio Software Suite (v 3.5) from Accelrys Software Inc. (San Diego, CA, USA).

Annexin V was supplied by BD Bioscience (Bristol, UK). Paclitaxel was from MP Biomedicals (Santa Ana, CA, USA). The mitochondrial uncoupler carbonyl cyanide 4-(trifluoromethoxy)-phenylhydrazone (FCCP), doxorubicin hydrochloride, propidium iodide, and DNase-free RNase A were obtained from Sigma (St. Louis, MO, USA). XTT and labeled UTP (TUNEL assay) were from Roche Diagnostics (Filderstadt, Germany). Caspase 3 substrate (Z-DEVD-R110) was from Bachem (Bubendorf, Switzerland) and the mitochondrial potential sensor 5,5′,6,6′-tetrachloro-1,1′, 3,3′-tetraethylbenzimidazolcarbocyanine iodide (JC-1) was purchased from Molecular Probes (San Diego, CA, USA). Antibody against the human proliferation antigen Ki-67 was from DakoCytomation (Glostrup, Denmark). 

### 4.2. Cell Lines

MDA-MB-231 and MCF-7 human breast cancer cell lines were purchased from the American Type Culture Collection (ATCC, Rockville, MD, USA) and maintained in Dulbecco’s modified Eagle medium (DMEM) supplemented with penicillin (50 units/mL), streptomycin (50 µg/mL), 1% non-essential amino acids, and 10% fetal bovine serum. The normal mammary epithelial cells (HME-1) were purchased from ATCC and cultured in mammary epithelial cell basal medium (MEBM) provided by Lonza (Basel, Switzerland). The other cell lines were from German Collection of Microorganisms and Cell Cultures (DSMZ, Braunschweig, Germany) and cultured as recommended by ATCC.

### 4.3. Cell Viability Analysis

The cell viability assay is based on the measurement of the optical density of the orange colored water-soluble formazan salt produced by viable cells with active mitochondrial dehydrogenase (mitochondrial reduction of tetrazolium salt). Cells were plated in 96-well microtiter plates and treated with the respective compounds for the specified times before incubating them with XTT labeling mixture at 37 °C [36,37,38]. The spectrophotometric absorbance was measured using a TECAN Infinite^®^ 200 PRO microplate reader (Männedorf, Switzerland) at 450 nm with a 630 nm-reference filter.

### 4.4. Cell Cycle Analysis

Following treatment with either of the polyhalogenated monoterpenes, doxorubicin (100 nM), or paclitaxel (100 nM), MDA-MB-231 cells were fixed in ice-cold 70% ethanol. The nuclei were stained with propidium iodide in RNase-containing buffer for 1 h at 37 °C [39] and analyzed by using flow cytometry (FACS Verse, BD Biosciences, San Jose, CA, USA). Cell doublets exclusion was performed before analysis to maintain the fidelity of the data. Further, cell cycle analysis was done with FlowJo software (TreeStar Inc., Ashland, OR, USA).

### 4.5. Analysis of Apoptosis 

Caspase 3 activity was analyzed using fluorometric detection of the cleaved tetrapeptide caspase 3/7 substrate Z-DEVD-R110. Briefly, the collected MDA-MB-231 cells were incubated with caspase 3/7 substrate (Z-DEVD-R110, 100 µM) in PBS for 1 h at 37 °C [36,37] and analyzed by flow cytometry (FACS Verse). To validate the contribution of caspase-dependent apoptosis in PPM1-induced cytotoxicity, MDA-MB-231 cells were pretreated with pancaspase inhibitor (z-VAD-fmk, 50 µM) (Calbiochem, San Diego, CA, USA) for 2 h before treatment with PPM1 (10 µM) for further 48 h and cell viability was analyzed using the XTT cell viability assay. Early and late apoptotic cells were sorted and quantified by flow cytometric analysis of Annexin V-FITC conjugate/propidium iodide double-stained cells [36]. The treated MDA-MB-231 cells were incubated with annexin V-FITC conjugate in binding buffer for 20 min in the dark. Annexin V binds with high affinity to phosphatidylserine exposed on the cell membranes as a sign of early apoptosis. Prior to acquisition, propidium iodide was added to identify the cells that had lost membrane integrity in the late stage of apoptotis. The percentages of the subdiploid G_1_ cells (subG_1_) with fragmented DNA were determined by using propidium iodide staining and FACS analysis of cells treated for 72 h [39].

### 4.6. Analysis of Mitochondrial Integrity

MDA-MB-231 cells were treated either with various concentrations of different polyhalogenated monoterpenes, or doxorubicin (100 nM) for 24 h. Changes in the mitochondrial membrane potential were determined using the voltage-sensitive lipophilic cationic probe, JC-1 sensor, which concentrates selectively within intact mitochondria to form multimeric J-aggregates emitting red fluorescence at 590 nm. By contrast, the monomeric form of the dye found in the cytosol of the cells upon leakage from damaged mitochondria emits green fluorescence at 527 nm, when excited at 490 nm. Treated cells were loaded with 10 µg/mL JC-1 dye in serum-free medium for 20 min at 37 °C [36,37] and analyzed by flow cytometry (FACS Verse, BD Biosciences, San Jose, CA). Appropriate compensation was set by using MDA-MB-231 cells treated with the mitochondrial uncoupler FCCP (50 µM, 6 h) as positive control.

### 4.7. Analysis of Histone H3 Phosphorylation

MDA-MB-231 cells were treated with respective compounds, fixed, permeabilized with methanol, and stained with antibodies against phospho-histone H3-S10 (1:50) and H3-T3 (1:200), both from Cell Signaling Technology (Danvers, MA, USA), followed by FITC-labeled secondary antibody, and flow cytometric analysis. DNA was counterstained with propidium iodide in buffer containing RNase. 

### 4.8. Western Blot Analysis

The effect of the polyhalogenated monoterpenes on signaling molecules was studied by Western immunoblot analysis of the total cell lysates. Briefly, the treated MDA-MB-231 cells were lysed in cold RIPA buffer (1.0% igepal CA-630, 0.5% sodium deoxycholate, and 0.1% sodium dodecyl sulphate (SDS) in PBS, pH 7.4) in the presence of protease and phosphatase inhibitors. Cell debris was removed by high-speed centrifugation and protein concentrations of the samples were determined using a Pierce BCA protein assay kit (Thermo Fisher Scientific, Waltham, MA, USA). Equal amounts of protein were separated by SDS-PAGE and electrophoretically transferred onto polyvinylidene fluoride (PVDF) membranes. Antibodies used are as follows: phospho-Aurora A^T288^/Aurora B^T232^/Aurora C^T198^, cyclin B_1_, p-CDK1^Y15^, CDK1, and p21^Waf1/Cip1^ (all 1:1000, Cell Signaling Technology), actin (1:500, Chemicon, Temecula, CA, USA), and Aurora kinase A (1:1000, IAK, BD Biosciences). Proteins were visualized with above antibodies, detected with corresponding horseradish peroxidase-coupled secondary antibodies and ECL^TM^ prime Substrate (GE Healthcare Life Sciences, Chicago, IL) using an Amersham^TM^ imager 600 from GE Healthcare Life Sciences. Expression of phosphorylated Aurora kinases A, B, C, total Aurora kinase A, and actin was quantified with an Amersham^TM^ imager 600 and ImageQuantTM TL 8.2 software (GE Healthcare Life Sciences, Chicago, IL, USA).

### 4.9. Human Tumor Xenografts as Experimental in Vivo Model

The study protocol complies with the Guide for the Care and Use of Laboratory Animals issued by US and European regulatory agencies. MDA-MB-231 cells were xenotransplanted onto the chick chorioallantoic membrane 8 days after fertilization. The next day, the xenografts were topically treated for 3 consecutive days with 20 µL of each compound. On day 12 after fertilization, the xenografts were collected, fixed, and paraffin-embedded for histological analysis. Serial sections (5 µm) were stained for the human proliferation antigen Ki-67. For the detection of apoptotic cells, DNA strand breaks were visualized by the terminal deoxynucleotidyl transferase dUTP nick-end labeling (TUNEL) method [36]. The sections were counterstained with hematoxylin and images were digitally recorded with an Axiophot microscope (Carl Zeiss, Göttingen, Germany) and a Sony MC-3249 CCD camera using Visupac 22.1 software (Carl Zeiss), and quantified using Gryphax® software (Jena, Germany). Avian embryos in this study were sacrificed prior to 7 days before hatching.

### 4.10. Statistical Analysis

All values are expressed as mean ± standard error of the mean (SEM). Statistical analysis was performed using the Newman-Keuls post-hoc test for multigroup comparisons by using Statistica software (StatSoft, Tulsa, OK, USA).

## References

[1] Torre L.A., Bray F., Siegel R.L., Ferlay J., Lortet-Tieulent J., Jemal A. (2015). Global cancer statistics, 2012. CA Cancer J. Clin..

[2] Siegel R.L., Miller K.D., Jemal A. (2018). Cancer statistics, 2018. CA Cancer J. Clin..

[3] Carey L., Winer E., Viale G., Cameron D., Gianni L. (2010). Triple-negative breast cancer: Disease entity or title of convenience?. Nat. Rev. Clin. Oncol..

[4] Lee E., McKean-Cowdin R., Ma H., Spicer D.V., Van Den Berg D., Bernstein L., Ursin G. (2011). Characteristics of triple-negative breast cancer in patients with a BRCA1 mutation: Results from a population-based study of young women. J. Clin. Oncol..

[5] Park J.H., Ahn J.H., Kim S.B. (2018). How shall we treat early triple-negative breast cancer (TNBC): From the current standard to upcoming immuno-molecular strategies. ESMO Open.

[6] Cabrita M.T., Vale C., Rauter A.P. (2010). Halogenated compounds from marine algae. Mar. Drugs.

[7] Fuller R.W., Cardellina J.H., Kato Y., Brinen L.S., Clardy J., Snader K.M., Boyd M.R. (1992). A pentahalogenated monoterpene from the red alga Portieria hornemannii produces a novel cytotoxicity profile against a diverse panel of human tumor cell lines. J. Med. Chem..

[8] Bucher C., Deans R.M., Burns N.Z. (2015). Highly selective synthesis of halomon, plocamenone, and isoplocamenone. J. Am. Chem. Soc..

[9] Vogel C.V., Pietraszkiewicz H., Sabry O.M., Gerwick W.H., Valeriote F.A., Vanderwal C.D. (2014). Enantioselective divergent syntheses of several polyhalogenated Plocamium monoterpenes and evaluation of their selectivity for solid tumors. Angew. Chem. Int. Ed..

[10] Sabry O.M., Goeger D.E., Valeriote F.A., Gerwick W.H. (2017). Cytotoxic halogenated monoterpenes from Plocamium cartilagineum. Nat. Prod. Res..

[11] Egorin M.J., Sentz D.L., Rosen D.M., Ballesteros M.F., Kearns C.M., Callery P.S., Eiseman J.L. (1996). Plasma pharmacokinetics, bioavailability, and tissue distribution in CD2F1 mice of halomon, an antitumor halogenated monoterpene isolated from the red algae Portieria hornemannii. Cancer Chemother. Pharmacol..

[12] Fuller R.W., Cardellina J.H., Jurek J., Scheuer P.J., Alvarado-Lindner B., McGuire M., Gray G.N., Steiner J.R., Clardy J., Menez E. (1994). Isolation and structure/activity features of halomon-related antitumor monoterpenes from the red alga Portieria hornemannii. J. Med. Chem..

[13] Tarhouni-Jabberi S., Zakraoui O., Ioannou E., Riahi-Chebbi I., Haoues M., Roussis V., Kharrat R., Essafi-Benkhadir K. (2017). Mertensene, a halogenated monoterpene, induces G_2_/M cell cycle arrest and caspase dependent apoptosis of human colon adenocarcinoma HT29 cell line through the modulation of ERK-1/-2, AKT and NF-kB signaling. Mar. Drugs.

[14] Andrianasolo E.H., France D., Cornell-Kennon S., Gerwick W.H. (2006). DNA methyl transferase inhibiting halogenated monoterpenes from the Madagascar red marine alga Portieria hornemannii. J. Nat. Prod..

[15] Schlama T., Baati R., Gouverneur V., Valleix A., Falck J.R., Mioskowski C. (1998). Total synthesis of (+/-)-halomon by a Johnson-Claisen rearrangement. Angew. Chem. Int. Ed..

[16] Sotokawa T., Noda T., Pi S., Hirama M. (2000). A three-step synthesis of halomon. Angew. Chem. Int. Ed..

[17] Jung M.E., Parker M.H. (1997). Synthesis of several naturally occurring polyhalogenated monoterpenes of the halomon class(1). J. Org. Chem..

[18] Afolayan A.F., Mann M.G., Lategan C.A., Smith P.J., Bolton J.J., Beukes D.R. (2009). Antiplasmodial halogenated monoterpenes from the marine red alga Plocamium cornutum. Phytochemistry.

[19] Antunes E.M., Afolayan A.F., Chiwakata M.T., Fakee J., Knott M.G., Whibley C.E., Hendricks D.T., Bolton J.J., Beukes D.R. (2011). Identification and in vitro anti-esophageal cancer activity of a series of halogenated monoterpenes isolated from the South African seaweeds Plocamium suhrii and Plocamium cornutum. Phytochemistry.

[20] De Ines C., Argandona V.H., Rovirosa J., San-Martin A., Diaz-Marrero A.R., Cueto M., Gonzalez-Coloma A. (2004). Cytotoxic activity of halogenated monoterpenes from Plocamium cartilagineum. Z. Naturforsch. C.

[21] De la Mare J.A., Lawson J.C., Chiwakata M.T., Beukes D.R., Edkins A.L., Blatch G.L. (2012). Quinones and halogenated monoterpenes of algal origin show anti-proliferative effects against breast cancer cells in vitro. Investig. New Drugs.

[22] Knott M.G., Mkwananzi H., Arendse C.E., Hendricks D.T., Bolton J.J., Beukes D.R. (2005). Plocoralides A-C, polyhalogenated monoterpenes from the marine alga Plocamium corallorhiza. Phytochemistry.

[23] Mann M.G., Mkwananzi H.B., Antunes E.M., Whibley C.E., Hendricks D.T., Bolton J.J., Beukes D.R. (2007). Halogenated monoterpene aldehydes from the South African marine alga Plocamium corallorhiza. J. Nat. Prod..

[24] Bianchini G., Balko J.M., Mayer I.A., Sanders M.E., Gianni L. (2016). Triple-negative breast cancer: Challenges and opportunities of a heterogeneous disease. Nat. Rev. Clin. Oncol..

[25] Lehmann B.D., Bauer J.A., Chen X., Sanders M.E., Chakravarthy A.B., Shyr Y., Pietenpol J.A. (2011). Identification of human triple-negative breast cancer subtypes and preclinical models for selection of targeted therapies. J. Clin. Invest..

[26] Hafner M., Niepel M., Chung M., Sorger P.K. (2016). Growth rate inhibition metrics correct for confounders in measuring sensitivity to cancer drugs. Nat. Methods.

[27] Dai J., Sultan S., Taylor S.S., Higgins J.M. (2005). The kinase haspin is required for mitotic histone H3 Thr 3 phosphorylation and normal metaphase chromosome alignment. Genes Dev..

[28] Flores M.L., Castilla C., Avila R., Ruiz-Borrego M., Saez C., Japon M.A. (2012). Paclitaxel sensitivity of breast cancer cells requires efficient mitotic arrest and disruption of Bcl-xL/Bak interaction. Breast Cancer Res. Treat..

[29] Katayama H., Brinkley W.R., Sen S. (2003). The Aurora kinases: Role in cell transformation and tumorigenesis. Cancer Metastasis Rev..

[30] Wang C., Youle R.J. (2009). The role of mitochondria in apoptosis. Annu. Rev. Genet..

[31] Fink S.L., Cookson B.T. (2005). Apoptosis, pyroptosis, and necrosis: Mechanistic description of dead and dying eukaryotic cells. Infect. Immun..

[32] Vitale I., Galluzzi L., Castedo M., Kroemer G. (2011). Mitotic catastrophe: A mechanism for avoiding genomic instability. Nat. Rev. Mol. Cell Biol..

[33] Fox D.T., Duronio R.J. (2013). Endoreplication and polyploidy: Insights into development and disease. Development.

[34] Gartel A.L., Tyner A.L. (2002). The role of the cyclin-dependent kinase inhibitor p21 in apoptosis. Mol. Cancer Ther..

[35] Masgras I., Carrera S., de Verdier P.J., Brennan P., Majid A., Makhtar W., Tulchinsky E., Jones G.D., Roninson I.B., Macip S. (2012). Reactive oxygen species and mitochondrial sensitivity to oxidative stress determine induction of cancer cell death by p21. J. Biol. Chem..

[36] El Gaafary M., Buchele B., Syrovets T., Agnolet S., Schneider B., Schmidt C.Q., Simmet T. (2015). An a-acetoxy-tirucallic acid isomer inhibits Akt/mTOR signaling and induces oxidative stress in prostate cancer cells. J. Pharmacol. Exp. Ther..

[37] El Gaafary M., Ezzat S.M., El Sayed A.M., Sabry O.M., Hafner S., Lang S., Schmiech M., Syrovets T., Simmet T. (2017). Acovenoside A Induces mitotic catastrophe followed by apoptosis in non-small-cell lung cancer cells. J. Nat. Prod..

[38] Schmidt C., Loos C., Jin L., Schmiech M., Schmidt C.Q., Gaafary M.E., Syrovets T., Simmet T. (2017). Acetyl-lupeolic acid inhibits Akt signaling and induces apoptosis in chemoresistant prostate cancer cells in vitro and in vivo. Oncotarget.

[39] Nicoletti I., Migliorati G., Pagliacci M.C., Grignani F., Riccardi C. (1991). A rapid and simple method for measuring thymocyte apoptosis by propidium iodide staining and flow cytometry. J. Immunol. Methods.

